# R-sulforaphane modulates the expression profile of AhR, ERα, Nrf2, NQO1, and GSTP in human breast cell lines

**DOI:** 10.1007/s11010-020-03913-5

**Published:** 2020-10-16

**Authors:** Barbara Licznerska, Hanna Szaefer, Violetta Krajka-Kuźniak

**Affiliations:** Department of Pharmaceutical Biochemistry, Poznan University of Medical Sciences, Poznań, Poland

**Keywords:** Sulforaphane, Nrf2, NQO1, GSTP, AhR, Breast cells

## Abstract

Our previous study showed remarkable differences in the effect of R-sulforaphane (R-SFN) on the expression of CYPs 19, 1A1, 1A2, and 1B1 in ER(+) MCF7, ER( −) MDA-MB-231, and non-tumorigenic immortalized MCF10A (8). This study aimed to evaluate the effect of R-SFN on phase II enzymes induction and expression of AhR, Nrf2, and ERα in the same breast cell lines. The results showed increased expression of GSTP as a result of treatment with R-SFN in breast cancer cells. An increased NQO1 transcript and protein levels were found in all breast cells, with the most significant increase in MCF7 cells. Similarly, the enhancement of Nrf2 expression was noticed in all tested cells. AhR gene transcript and protein were decreased in MCF7 cells. In MDA-MB-231, increased AhR mRNA was not confirmed at the protein level. No differences were found in the expression of ERα. Overall, the results of the present study extended our earlier suggestions on the possible interference of R-SFN with estrogens homeostasis in breast cancer cells differing in ERα status, as well as in non-tumorigenic immortalized breast epithelial cells. While some of R-SFN effects might be beneficial and useful in breast cancer prevention, the others, particularly GSTP induction, may lead to adverse effects.

## Introduction

Breast cancer is a leading cause of death and the most common malignancy among women worldwide. Prolonged exposure to elevated levels of estrogens is considered major breast cancer risk factors. Estrogens may contribute to breast cancer development in two ways (i) by induction of proliferation via estrogen receptor (ER), (ii) by a generation of reactive metabolites of estrogens such as estrogen quinones as well as the formation of oxygen free radicals [1]. Reactive metabolites are produced in reactions catalyzed by different isoforms of cytochrome P450, particularly the CYP1 family [2, 3].

The expression of *CYP1* genes is regulated by the aryl hydrocarbon receptor (AhR). This ligand-activated transcription factor belongs to the basic 40 helix-loop-helix (bHLN)/Per-Arnt-Sim family of “sensors” of foreign and endogenous signals [4]. AhR is present in the cytoplasm in a latent complex with two heat-shock-protein (Hsp90s) and related chaperones [5]. Upon ligand binding, AhR translocates to the nucleus, heterodimerizes with the AhR nuclear translocator, and binds xenobiotic response elements (XREs) in the regulatory region of many genes, including Phase I and II xenobiotic-metabolizing enzymes from the AhR gene battery [6]. AhR not only activates the expression of genes metabolizing estrogens but also promotes the degradation of estrogen receptor α (ERα) [7]. Therefore, it can be assumed that the modulation of AhR and, subsequently, the expression of genes controlled by this receptor may depend on breast cells' estrogen receptor status. In this regard, our previous study showed that the effect of naturally occurring R-sulforaphane (R-SFN, a synonym of l-sulforaphane), (−)1-isothiocyanato-4R-(methylsulfinyl)-butane, on CYP19, CYP1A1, 1A2, and 1B1 expression differed significantly between ERα(+) and ERα(−) breast cells [8]. Moreover, an increased level of CYP1A2 and decreased level of CYP1B1 expression were found in non-tumorigenic immortalized MCF10A cells. Overall, this study showed that R-SFN might affect the expression of P450s involved in estrogen metabolism, particularly CYP19, which converts androgens to estrogens in the target tissue and is the first important factor implicated in breast carcinogenesis. Besides, racemic SFN and its derivatives inhibited CYP1A1 and CYP1A2 enzyme activity in MCF7 breast cancer cells [9]. SFN is a well-known inducer and activator of phase II enzymes. In this regard, R-SFN increased hepatic glutathione S-transferase and NAD(P)H: quinone oxidoreductase 1 (NQO1) and up-regulated GSTα, GSTµ, and NQO1 protein levels [10, 11]. These enzymes detoxify carcinogenic metabolites, including reactive estrogen forms, thus preventing carcinogenesis initiation.

The expression of phase II enzymes is controlled by the nuclear factor erythroid 2-related factor 2 (Nrf2), a transcription factor. Upon activation, Nrf2 translocates into the nucleus and binds to ARE sequence, which results in increased expression of antioxidant and cytoprotective enzymes included that mentioned above. While induction of phase II enzymes by SFN was widely described in many tissues and cells, e.g., hepatocytes or colon cells, the data showing its influence in breast cells are scanty and limited to non-tumorigenic epithelial breast cells. Nrf2 signaling is positively modulated by the AhR but inhibited by estrogen receptor alpha (ERα). Such cross-talk through altered p300 recruitment to Nrf2-regulated target genes was shown in ER(+) MCF7 breast cancer cells treated with racemic SFN, a combination of AhR and ERα activator, and 17β-estradiol (E2) [12]. The results of this study on the effect of racemic SFN on untransformed human colon epithelial cells and colorectal cancer cells indicated a different impact of SFN on Nrf2 expression and Nrf2-dependent signaling pathways in these two cell types [13]. Therefore, such an effect can also be expected in breast epithelial cells. Moreover, it has to be pointed out that most of the investigations on SFN were performed using its racemic form, while in nature, only R-SFN occurs.

This study aimed to evaluate the effect of this SFN enantiomer on phase II enzymes AhR, Nrf2, and ERα expression in breast cancer cell lines differing in ERα status (i.e., ER(+) MCF7, ER(−) MDA-MB-231) and non-tumorigenic immortalized epithelial breast cell line (MCF10A).

## Materials and methods

### Chemicals

The main supplier of chemicals was Sigma-Aldrich, in particular for (R)-sulforaphane: (−)1-isothiocyanato-4R-(methylsulfinyl)-butane (CAS 14825-10-3), antibiotics solution (penicillin/streptomycin/amphotericin B), bovine serum albumin, dimethylsulfoxide (DMSO), dithiothreitol, Dulbecco’s Modified Eagle’s Medium (DMEM), fetal bovine serum (FBS), hydrocortisone, insulin, epidermal growth factor (EGF), horse serum, RIPA buffer, trypsin, Tris, and tRNA from *E. coli*. For more details, e.g., the concentrations of chemicals see our previous publications [8]. Primary antibodies against Nrf2, NQO1, AhR, ERα, β-actin, lamin, and secondary antibodies were supplied by Santa Cruz Biotechnology. The primary antibody against GSTP1 was obtained from LabAs. Protease inhibitor tablets were bought from Roche. R-SFN was dissolved in DMSO at a concentration of 100 mmol/L and stored at -20 °C.

### Cell culture and treatment

Breast cancer cell lines MCF7, MDA-MB-231, and MCF10A were obtained from the European Collection of Cell Cultures. The cells were cultured in standard conditions: density up to 70% confluence, 5% CO_2,_ and 95% air, in DMEM with proper supplements indicated previously. After a 24 h pre-incubation period, the cells were treated with R-SFN at the doses of 5 μM (for all cell lines), 10 μM (for MCF10A cells), or 20 μM (for MCF7 and MDA-MB-231 cells), which were selected based on cytotoxicity assay [8]. The incubation was continued for the subsequent 72 h. Simultaneously, control cells were treated with DMSO (vehicle) at the concentration not exceeding 0.1%.

### Preparation of nuclear and cytosolic extracts

The nuclear and cytosolic extracts were prepared using a Nuclear/Cytosol Fractionation Kit (BioVision Research) according to the manufacturer’s instructions. Briefly, cells were collected by centrifugation at 600×*g* for 5 min at 4 °C. Pellets were resuspended in ice-cold cytosol extraction buffer containing DTT and protease inhibitors. After incubation in an ice bath for 10 min, the samples were centrifuged for 5 min at 16,000×*g* at 4 °C to collect the cytosolic fraction. The supernatants (cytosolic fractions) were transferred to clean tubes. The pellets were resuspended in ice-cold nuclear extraction buffer containing DTT and protease inhibitors and incubated again in an ice bath for 40 min with vortex mixing for 15 s every 10 min. The lysed suspensions of nuclei were then centrifuged at 16,000×*g* at 4 °C for 10 min, and the collected nuclear extracts were stored at − 70 °C.

### Real-time PCR

The GenElute Mammalian Total RNA Miniprep Kit (Sigma) was used for total RNA isolation conducted according to the manufacturer’s recommendations. Total RNA was subjected to reverse transcription using the RevertAid First Strand cDNA Synthesis Kit (Fermantas). The reaction was followed by quantitative real-time PCR in triplicate using Maxima SYBR Green/ROX qPCR Master Mix (Fermentas) and BioRad Chromo4 system. The protocol started with 5 min enzyme activation at 95 °C, followed by 40 cycles of 95 °C for 15 s; 56 °C for 20 s; 72 °C for 40 s and final elongation at 72 °C for 5 min. The melting curve analysis verified the product size. Experiments were normalized for expression of the TATA-box binding protein (TBP) and porphobilinogen deaminase (PBGD). The Pfaffl relative method was used for fold-change quantification. The following primers were used: forward/reverse—AhR (5′ACAGATGAGGAAGGAACAGAG3′/ 5′CTTGCTTAGAGTGGATGTGG3′); ERα (5′GGGTGGCAGAGAAAGATTG3′/ 5′TATAGTCGTTATGTCCTTGAATAC3′); GSTP (5′GCAAATACATCTCCCTCATC3′/ 5′AGGTTGTAGTCAGCGAAG3′); NQO1 (5′CAATTCAGAGTGGCATTC3′/ 5′GAAGTTTAGGTCAAAGAGG3′); Nrf2 (5′ATTGCTACTAATCAGGCTCAG3′/ 5′GTTTGGCTTCTGGACTTGG3′); PBGD (5′TCAGATAGCATACAAGAGACC3′/ 5′TGGAATGTTACGAGCAGTG3′); TBP (5′GGCACCACTCCACTGTATC3′/5′GGGATTATATTCGGCGTTTCG3′).

### Western blot analysis

Immunoblot assay was used to determine the level of AhR, ERα, Nrf2, GSTP, or NQO1 proteins. Whole-cell lysates (AhR and ERα), nuclear extracts (Nrf2), or cytosolic extracts (Nrf2, GSTP or NQO1) (100 μg) were separated on either 7.5%, 10% or 12% SDS-PAGE slab gels, and the proteins were transferred to nitrocellulose membranes [14]. After blocking with 10% skimmed milk, the proteins were probed with goat polyclonal AhR, goat polyclonal ERα, rabbit polyclonal Nrf2, mouse polyclonal GSTP, goat polyclonal NQO1, rabbit polyclonal β-actin, and rabbit polyclonal lamin antibodies. The β-actin and lamin proteins were used as an internal control. Alkaline phosphatase-labeled anti-rabbit IgGs, anti-mouse IgGs, and anti-goat IgGs were used as secondary antibodies in the staining reaction. The amount of immunoreactive products in each lane was determined using Quantity One software (BioRad). Values were calculated as relative absorbance units (RQ) per mg of protein.

### Statistical analysis

The one-way ANOVA tool was utilized for statistical analysis. The statistical significance between the experimental groups and their respective controls was assessed by Dunnett’s post hoc test at *p* < 0.05.

## Results

### The effect of R-sulforaphane on the expression of phase II enzymes

**Fig. 1:**
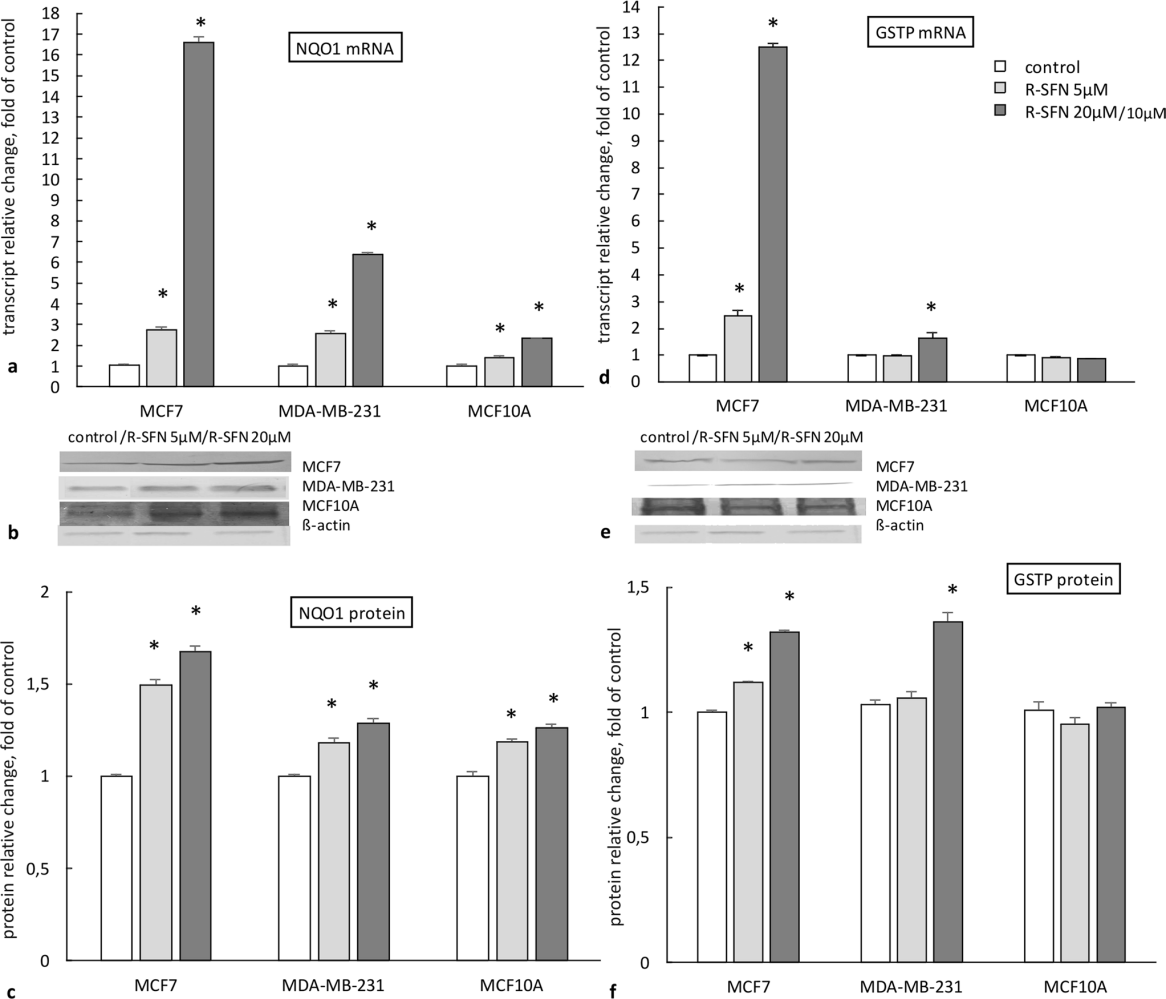
The effect of 72 h incubation with *R-*sulforaphane (R-SFN) on the level of the *NQO1* transcript (**a**) and protein (**c**), and the *GSTP* transcript (**d**) and protein (**f**) in MCF7, MDA-MB-231, and MCF10A cell lines. **b** and **e** show representative blots for NQO1 (**b**) and GSTP (**e**) proteins. The values were calculated as a relative change in transcript or protein level in comparison with control cells (expression equals 1). The mean values ± SEM from three independent experiments performed in triplicate are presented. *Mean values were significantly different from the control cells (*p* < 0.05)

Figure 1d–f shows the effect of R-SFN on the expression of the *GSTP* gene. While significant differences between tested cell lines, with the highest mRNA transcript level in MCF7 cells, were observed, there were no differences between the level of GSTP protein in ERα(+) and ERα(−) breast cancer cells. GSTP, both mRNA transcript and protein levels, were unaffected in MCF10A non-tumorigenic cells breast cells.

More significant differences between the cell lines were observed in the case of the *NQO1* gene. R-SFN treatment increased the most the *NQO1* transcript and protein levels in MCF7 cells. R-SFN also increased the expression of this gene in MDA-MB-231 to an extent similar to that observed in MCF10A cells (Fig. 1a–c).

### The effect of R-sulforaphane on the expression of Nrf2

**Fig. 2:**
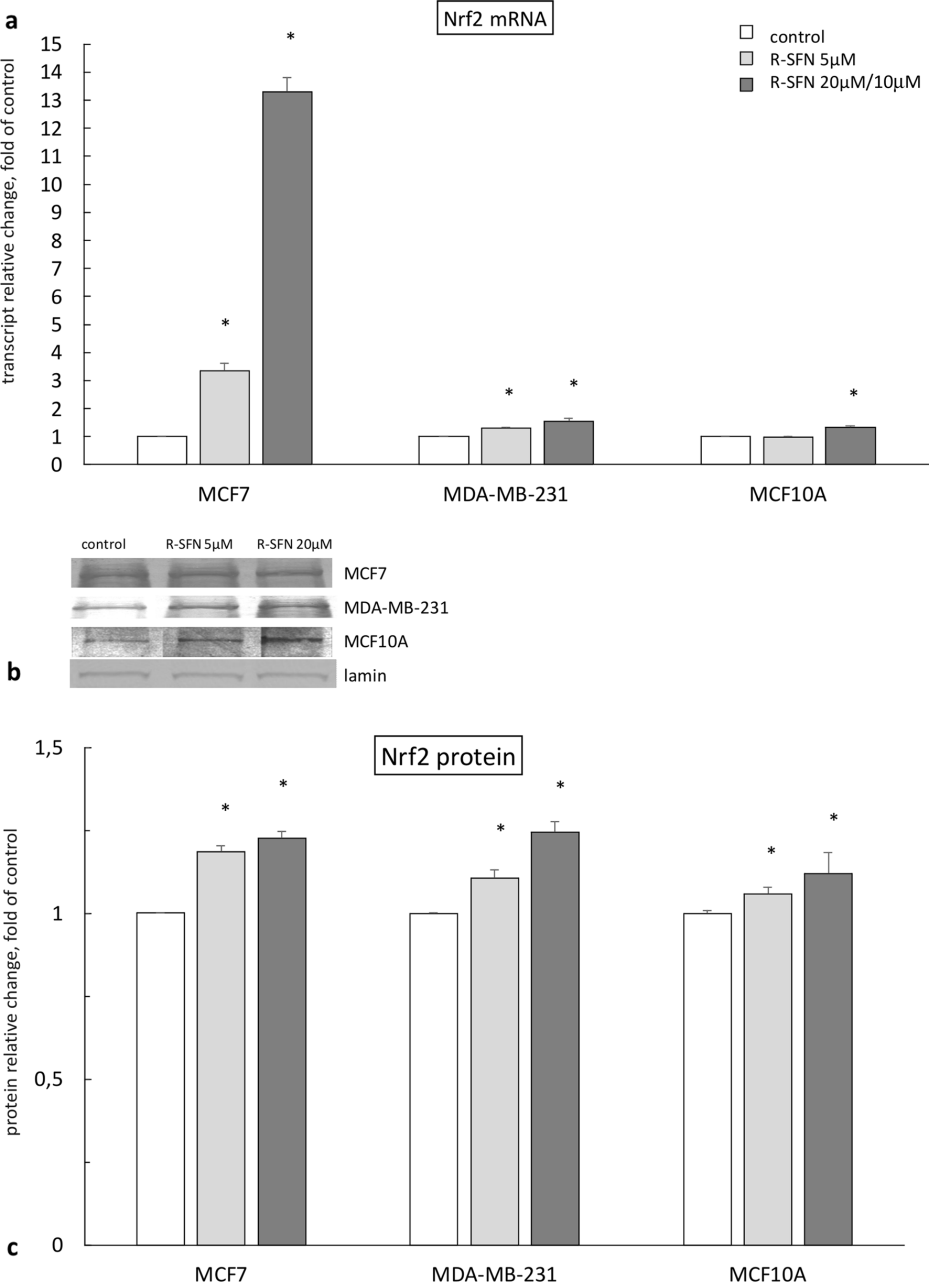
The effect of 72 h incubation with *R-*sulforaphane (R-SFN) on the level of the *Nrf2* transcript (**a**) and protein (**c**) in MCF7, MDA-MB-231, and MCF10A cell lines. **b** Representative blots for Nrf2 protein. The values were calculated as a relative change in transcript or protein level in comparison with control cells (expression equals 1). The mean values ± SEM from three independent experiments performed in triplicate are presented. *Mean values were significantly different from the control cells (*p* < 0.05)

A similar trend, as observed in the case of the *GSTP* gene, was found in the impact of R-SFN on *Nrf2* gene expression. The highest increase of Nrf2 transcript was observed in MCF7 cells. However, this observation was not confirmed at the protein level, where no differences between MCF7 and MDA-MB-231 cells were observed. A slightly lower increase of the Nrf2 protein level was found in MCF10A cells (Fig. 2).

### The effect of R-sulforaphane on the expression of AhR

**Fig. 3:**
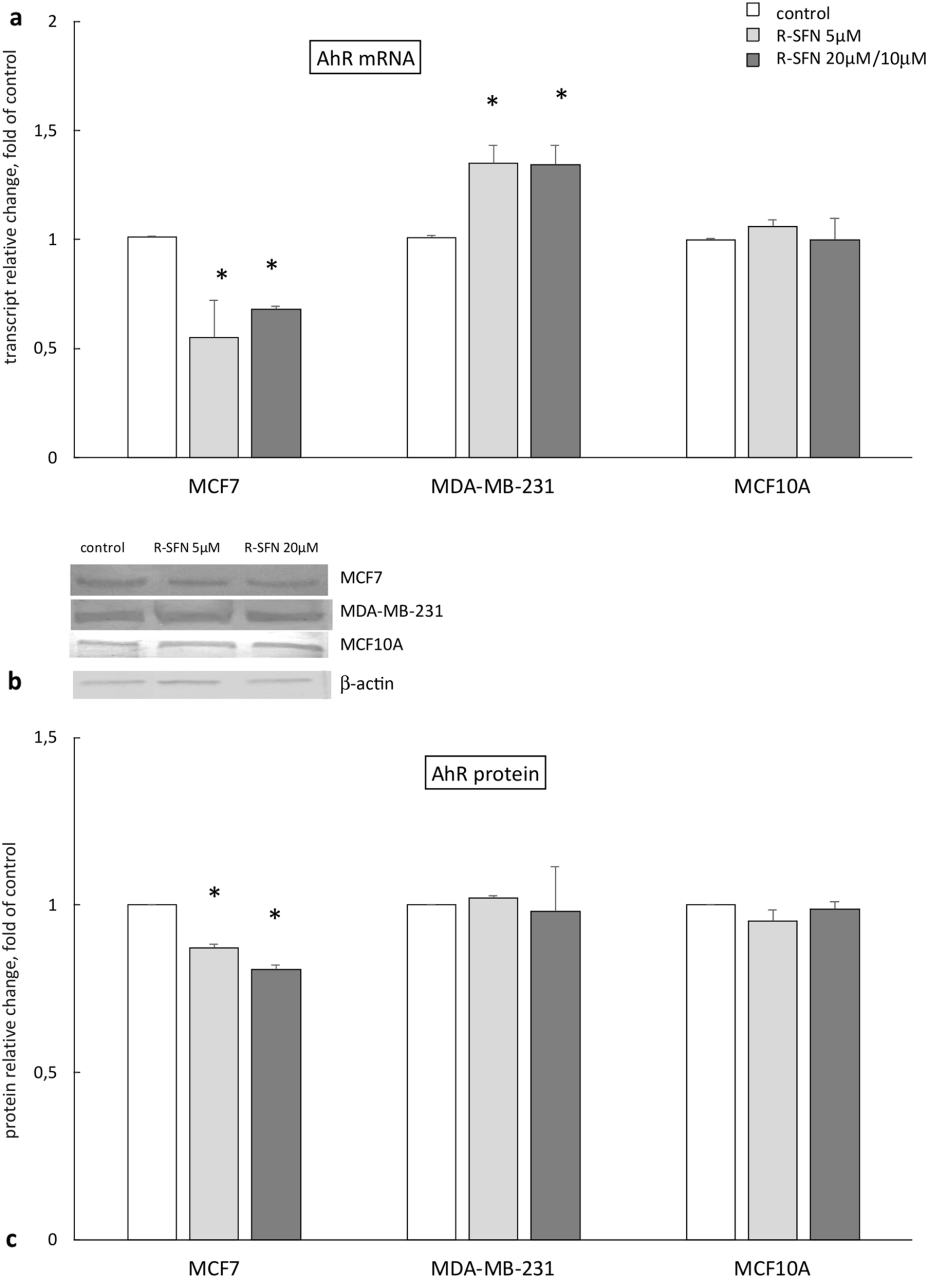
The effect of 72 h incubation with *R-*sulforaphane (R-SFN) on the level of the *AhR* transcript (**a**) and protein (**c**) in MCF7, MDA-MB-231, and MCF10A cell lines. **b** Representative blots for AhR protein. The values were calculated as a relative change in transcript or protein level in comparison with control cells (expression equals 1). The mean values ± SEM from three independent experiments performed in triplicate are presented. *Mean values were significantly different from the control cells (*p* < 0.05)

Striking differences in the *AhR* transcript levels were observed as the effect of treatment with R-SFN between MCF7 and MDA-MB-231 cells (Fig. 3). While in ERα (+) cells, decreased *AhR* mRNA level was observed, in ERα (-) MDA-MB-231 cells, an increased transcript level was found.

However, these differences in *AhR* gene expression were not confirmed at the protein level. While in MDA-MB-231 and MCF10A cells AhR protein level was unchanged, in MCF7 cells, in concert with transcript protein level, was diminished in comparison with untreated control.

### The effect of R-sulforaphane on the expression of ERα

**Fig. 4:**
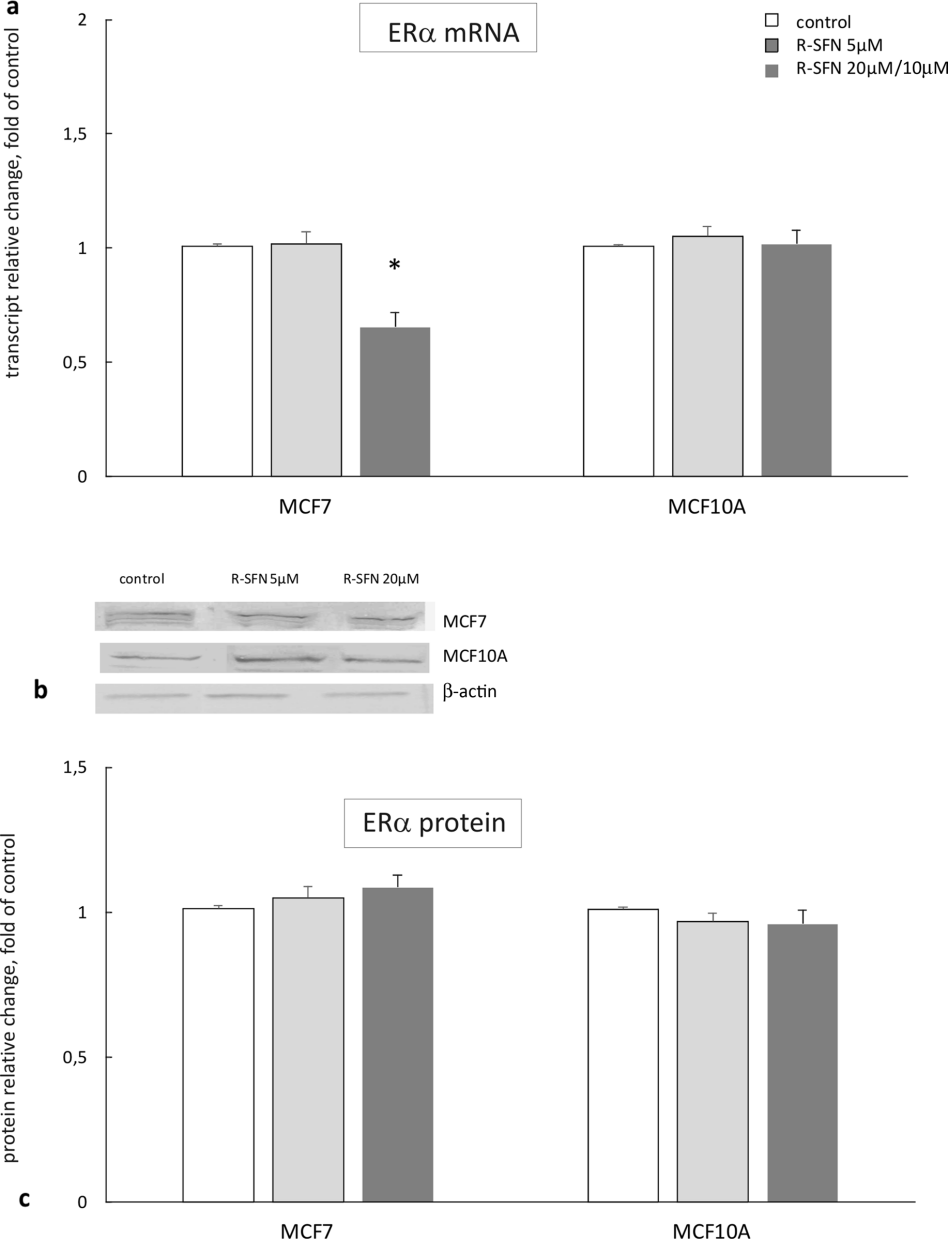
The effect of 72 h incubation with *R-*sulforaphane (R-SFN) on the level of the *ERα* transcript (**a**) and protein (**c**) in MCF7 and MCF10A cell lines. **b** Representative blots for *ERα* protein. The values were calculated as a relative change in transcript or protein level in comparison with control cells (expression equals 1). The mean values ± SEM from three independent experiments performed in triplicate are presented. *Mean values were significantly different from the control cells (*p* < 0.05)

R-SFN treatment at the concentration of 20 µM decreased *ERα* gene transcript in MCF7 cells but did not affect transcription in non-tumorigenic MCF10A cells. However, the ERα protein was not changed in both cell lines as a result of R-SFN treatment (Fig. 4).

## Discussion

The oxidative metabolism of estrogens plays a crucial role in the initiation of estrogen-induced breast cancer. The generation of reactive electrophiles such as catechol estrogen-3,4-quinones is the result of disruption of estrogen homeostasis, e.g., upregulation of CYP19 and CYP1B1 on the one hand and downregulation of CYP1A1 on the other. Catechol estrogen-3,4-quinones can be detoxified by phase II enzymes such as GST or NQO1 [1]. Cruciferous vegetable consumption is linked with a lower risk of breast cancer, which at least in part might be related to the interference of their active ingredients with estrogen homeostasis.

In this regard, our previous study has shown that R- SFN, a common ingredient of broccoli, modified the expression of CYP19, CYP1A1, 1A2, 1B1 in breast cell lines differing in ER status. Expression of the CYP1 family of genes is controlled by AhR, which acts as a transcription factor but may also initiate a reduction of estrogen signaling.

The results of this study did not clearly confirm the involvement of AhR-ER interaction in response to R-SFN breast cells treatment. AhR transcript and protein levels were reduced only in ER(+) MCF7 cells. In contrast, in MDA-MB-231 ER(−) cells, AhR transcript level was increased but not confirmed on the protein level. A similar effect, i.e., a decreased level of mRNA and unchanged protein, was observed in the effect of R-SFN on the expression of ERα in MCF7 cells. Thus, it appears that R-SFN influences the expression of these receptors through both transcriptional and post-transcriptional mechanisms. There are many processes between transcription and translation, which affect the protein level. The half-life of different proteins can vary from minutes to days, whereas the degradation rate of mRNA would fall within a much tighter range, 2–7 h for mammalian mRNAs vs. 48 h for protein [15].

Overall, in contrast to the results of our previous study, where the possible interplay between indole-3-carbinol (I3C), the product of its condensation diindolylmethane (DIM) and estrogens, on the expression of CYP1A1 and 1B1 genes in MCF7 cells was confirmed, the results of the current study do not support such interplay in case of R-SFN. However, similarly as in the case of I3C and DIM, the inverse interplay of R-SFN and estrogens might potentially occur in non-tumorigenic immortalized MCF10A cells [14].

The most interesting observation of this study is the induction by R-SFN phase II enzymes GSTP and NQO1. While the induction of these enzymes by racemic SFN was the subject of earlier studies in non-tumorigenic breast epithelial cells [16, 17], there is no data on the effect of SFN on the expression of their genes in breast cancer ER(+) and ER( −) cells. Moreover, in MCF10A cells the increased expression of *GSTA1* transcript level was described only [16]. Our study showed increased both mRNA and protein of GSTP as a result of treatment in MCF7 and MDA-MB-231 breast cancer cells. While the reduced expression of GSTA1 is associated with increased breast cancer primarily among women with lower consumption of cruciferous vegetables and among current smokers [18], the increased expression of GSTP1 predicts poor pathological complete response to neoadjuvant chemotherapy in ER-negative breast cancer [19]. Moreover, a more recent study by Louie et al. [20] demonstrated that GSTP1 is a driver of triple-negative breast cancer (TNBC) cell metabolism and pathogenicity and might be considered as a novel therapeutic target for this type of breast cancer. Therefore, the increased expression of GSTP as a result of treatment with R-SFN, particularly in MDA-MB-231 cells, has to be considered as an adverse effect [21].

On the other hand, treatment with R-SFN increased the expression of the *NQO1* gene both in breast cancer (MCF7 and MDA-MB-231) and non-tumorigenic breast cells. The highest expression was observed in ER(+) MCF7 cells. NQO1 catalyzes the reduction of catechol estrogen-3,4-quinones back to their catechol estrogens and thus exert chemoprotective response. Differently, NQO1-mediated two-electron reduction converts certain quinone compounds such as mitomycin C or β-lapachone to cytotoxic agents, leading to cell death. It is well-known that NQO1 is expressed at high levels in numerous human cancers, including breast, colon, cervix, lung, and pancreas, as compared with normal tissues. Thus, tumors can be preferentially damaged relative to normal tissue by cytotoxic quinone drugs [22]. Therefore, the induction of NQO1 by R-SFN might be considered beneficial in two different ways. In non-tumorigenic and partly MCF7 cells, which represent the early stage of breast carcinogenesis, R-SFN may protect against cancer initiation and progression, respectively, while in MDA-MB-231 may support therapy with certain chemotherapeutics.

Induction of phase II enzymes usually occurs via Nrf2- dependent mechanisms. In concert with the induction profile of NQO1, the Nrf2 expression was increased as a result of treatment with R-SFN in all tested cells, with the highest increase in MCF7 cells. There is no doubt that Nrf2 can protect against cancer initiation or promotion by protecting against genotoxic insults. On the other hand, it is also well established that a great number of tumors exhibit enhanced Nrf2 activity, which may contribute to a malignant phenotype and increased chemo-resistance. However, it was also shown that pharmacological activation of Nrf2 is distinct from genetic activation and does not provide a growth or survival advantage to tumor cells [23]. Therefore, it is possible that induction of Nrf2 by R-SFN should not interfere with conventional therapy but rather support it.

Overall, the results of the present study extended our earlier suggestions on the possible interference of R-SFN with estrogens’ homeostasis in breast cancer cells differing in ER status as well as in non-tumorigenic immortalized breast epithelial cells. While some of R-SFN effects might be beneficial and useful in breast cancer prevention, the others, particularly GSTP induction, may lead to adverse effects. No clear cross-talk between AhR-ER and Nrf2 has been established.
